# Study of Interactions between Metallothionein and Cisplatin by using Differential Pulse Voltammetry Brdickás reaction and Quartz Crystal Microbalance

**DOI:** 10.3390/s90301355

**Published:** 2009-02-26

**Authors:** Dalibor Huska, Ivo Fabrik, Jiri Baloun, Vojtech Adam, Michal Masarik, Jaromir Hubalek, Anna Vasku, Libuse Trnkova, Ales Horna, Ladislav Zeman, Rene Kizek

**Affiliations:** 1 Department of Chemistry and Biochemistry, Faculty of Agronomy, Mendel University of Agriculture and Forestry, Zemedelska 1, CZ-613 00 Brno, Czech Republic; 2 Department of Animal Nutrition and Forage Production, Faculty of Agronomy, Mendel University of Agriculture and Forestry, Zemedelska 1, CZ-613 00 Brno, Czech Republic; 3 Department of Pathological Physiology, Faculty of Medicine, Masaryk University, Kamenice 5, CZ-625 00 Brno, Czech Republic; 4 Department of Microelectronics, Faculty of Electrical Engineering and Communication, Brno University of Technology, Udolni 53, CZ-602 00 Brno, Czech Republic; 5 Department of Chemistry, Faculty of Science, Masaryk University, Kamenice 5, CZ-625 00 Brno, Czech Republic; 6 Tomas Bata University, T.G. Masaryka 275, CZ-762 72 Zlin, Czech Republic

**Keywords:** Cancer, Metallothionein, Cisplatin, Protein-Drug Interaction, Voltammetry, Brdickás reaction, Quartz Crystal Microbalance

## Abstract

Treatment strategies for tumour diseases are progressively focusing on personalization of medicine. However, this focus requires methods revealing the early general biological mechanisms, including the formation anti-cancer drugs’ resistance. The low molecular mass protein metallothionein is thought to be the crucial for the formation of resistance in tumour treatment based on the platinum-cytostatics. The interactions between metallothionein (MT) and cisplatin were determined by the adsorptive transfer stripping technique coupled with the differential pulse votlammetry Brdickás reaction. The signals related to the MT-cisplatin complex appeared at −0.9 V. The formation of this complex depended on the time of interaction between cisplatin and MT. The complex formation was consequently confirmed by quartz crystal microbalance analyses. The formation of this complex was detectable even after a 20 s long interaction. Moreover, we detected presence of MT-cisplatin complex in the blood of male rats treated with this drug.

## 1. Introduction

The metallothioneins (MT) are the group of the low molecular mass (about 6.5 kDa) heavy metal binding proteins. MTs are a family of proteins with a large degree of sequence homology, which are found in bacteria, fungi, plants, and animal species. They consist of approximately 65 amino acids with an extremely high occurrence of cysteine residues. The metal binding domain of MT consists of 20 cysteine residues juxtaposed with the basic amino acids (lysine and arginine) arranged in two thiol-rich sites [1–3]. Thiol-rich sites bind heavy metals ions with different affinity [4]. Due to their main ability of binding heavy metals MT play a crucial role in the maintenance of homeostasis of the essential metals ions. It was found that MT synthesis increased with the increase of essential as well as toxic heavy metals ions. Thus, MT levels are considered a biochemical marker of acute poisoning by heavy metals ions [5–8]. Besides this metallothionein gene can be inserted into plants to enhance their ability to withstand higher concentrations of heavy metal ions in the environment [9,10]. From a clinical point of view, MT appear to be markers of the enhanced cell proliferation and probably play a considerable role in the formation of the resistance to platinum-based-drug therapy [3,11].

For the determination of MT the various techniques and methods are employed, including the most commonly used immunochemistry [12–15]. Spectrometric [13,15,16] and chromatographic ones [17–19] can also be used, although electrochemical methods are among the most sensitive, low cost and easy to use, with detection limits below nM [20]. In spite of the large battery of the techniques available detection of MT in the blood or blood serum is still a difficult task. Recently we have shown that serum MT levels of patients with a tumour disease were significantly enhanced [11,21,22]. In addition, the enhanced MT level was associated with the progression of the malignant disease. There are several other authors and research groups interested in the study of the relationship between MT with tumour disease progression. Recently, papers reporting the role of metallothionein in breast tumours [23,24], thyroidal tumours [25,26], prostate gland tumours [27–29], head and neck tumours [30] and liver tumours [31,32] were published.

**Figure 1.**
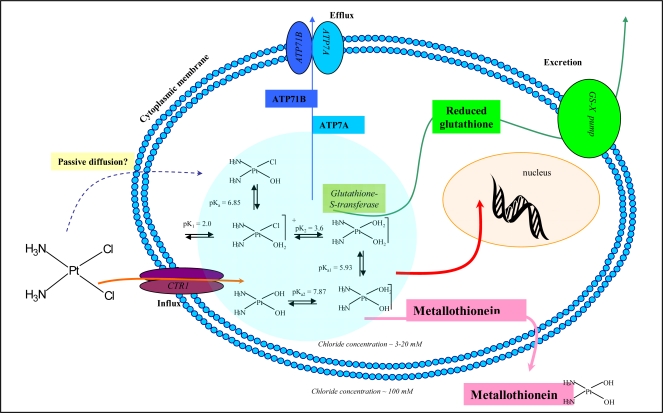
A simplified scheme of the possible reactions in a cell after cisplatin entry. Cisplatin can enter a cell through channels (e.g. CTRI) or also by the passive diffusion. In the cell, aqua complexes of cisplatin are able to react with DNA especially due to changes in chloride ion concentrations. However, cisplatin concentration in a cell is lowered by the active efflux through ATP pump and by reactions with glutathione and metallothionein. The scheme was proposed and modified according to [37,38].

Platinum complexes play an important role in the chemotherapy of the various malignancies. In spite of the fact that the first platinum complex, cisplatin, was first used almost forty years ago, the exact mechanism of action remains unclear [33]. A platinum based cytostatic drug can coordinate to N7 of two neighbouring guanine and/or adenine bases, in the same or in opposite DNA strands, as this nitrogen does not form H bonds with the other bases [34]. The transport of cisplatin into a cell is the other crucial step. The cells treated with cisplatin protect themselves against it with the various mechanisms. Efflux of this drug by ATP pumps is one of them. Another very important mechanism of cisplatin resistance is based on the action of the enzyme glutathione-*S*-transferase (GST). GST transports cisplatin to reduced glutathione (GSH) and the GS-cisplatin complex is excreted out of cells. In addition to the above mentioned mechanisms, MT can interact with cisplatin too [12,35,36]. MT binds molecules of cisplatin that are consequently excreted out of a cell (Figure 1). However the exact role of MT in formation of resistance on platinum based-cytostatics remains unclear.

**Figure 2.**
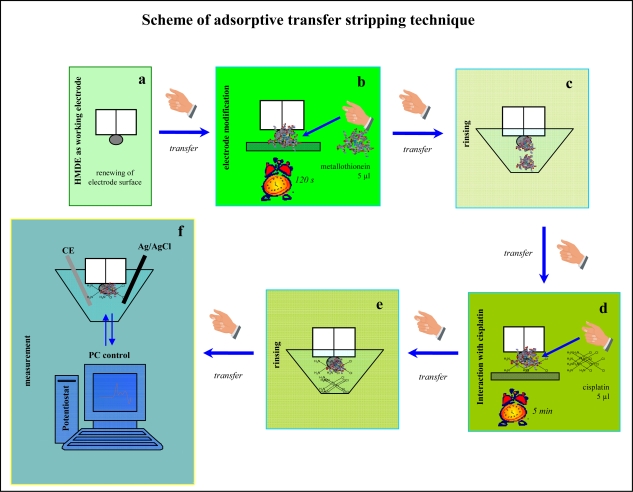
The principle of the adsorptive transfer stripping (AdTS) technique is based on the strong adsorption of the analyte on the electrode surface at the open electrode circuit. (a) A hanging mercury drop electrode surface is renewed. (b) The renewed electrode is immersed in a drop containing a target molecule (metallothionein) to be adsorbed on the surface of this electrode. (c and d) The excess analyte is rinsed from the surface of the working electrode in the buffer and the electrode is transferred to the solution containing a compound with which molecules adsorbed in previous step interacts. After the other rinsing step (e) the adsorbed complex is detected in the presence of supporting electrolyte (f) [35,36,46,50].

As a consequence of the use of platinum based cytostatic drugs in the treatment of tumour diseases, it became necessary not only to detect them in the biological samples but also to investigate their interactions with intracellular peptides and proteins. The study of these interactions is still difficult task. Various spectrometric techniques such as the mass spectrometry, atomic absorption spectrometry or UV-VIS spectrometry, coupled with appropriate separation techniques, are used for this purpose and have been reviewed by Timerbaev *et al.* [39]. Computational approaches are used too [40–42] The adsorptive transfer stripping technique (AdTS) has many use advantages for studying heavy metal-protein interactions [5,11,20,22,35–37,43–50]. A scheme of AdTS is shown in Figure 2. Besides AdTS, the quartz crystal microbalance represents another very promising tool in interaction studies [51–54]. The main aim of this work was to study the interaction of MT with cisplatin by using AdTS coupled with differential pulse voltammetry and a quartz crystal microbalance.

## 2. Results and Discussion

### 2.1. Metallothionein-cisplatin interaction

**Figure 3.**
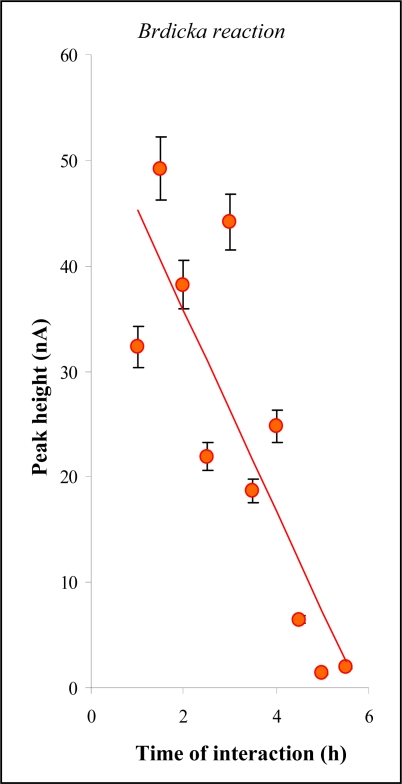
The changes of catalytic Cat2 peak height during MT (10 μg mL^−1^) interaction with cisplatin (100 μg mL^−1^), n = 5.

We have studied primarily the interaction of MT with cisplatin by Brdickás reaction. This method was used for decades for the determination of peptides and proteins rich in thiol moieties [36,49,55–57]. Coupling of Brdickás reaction with AdTS seems to be a very promising tool for the detection of compounds rich in thiols [49]. The complexation of the cisplatin and MT and the subsequent reduction of the complexes at the electrode is one of possible modifications of Brdickás reaction to determine MT [58,59]. It could be suggested that cisplatin-MT complexes can be easily formed. Our experiments were carried out accordingly. MT (5 μL, 10 μg mL^−1^) was placed on Parafilm. A hanging mercury drop electrode (HMDE) was subsequently immersed into this drop. MT was accumulated for 120 s onto the surface of immersed HMDE. After the accumulation step, HMDE was rinsed. MT has very good affinity to HMDE surface and forms an organised layer on the HMDE surface after a short time period [50]. The MT-modified HMDE was subsequently transferred into a microlitre volume of cisplatin. After a certain time interval, the electrode was rinsed and transferred into the supporting electrolyte, where the electrochemical detection was carried out. MT itself gave the typical Brdickas’ signals as RS2Co, Cat1, Cat2 and Cat3 [49,56]. The changes in Cat2 height with the increasing time of the interaction with cisplatin are shown in Figure 3. The Cat2 peak was decreasing with the increasing time of interaction. After 5 min long interaction, the decrease was higher than 95 %.

### 2.2. Electrochemical study of metallothionein-cisplatin complexes in rat blood

**Figure 4.**
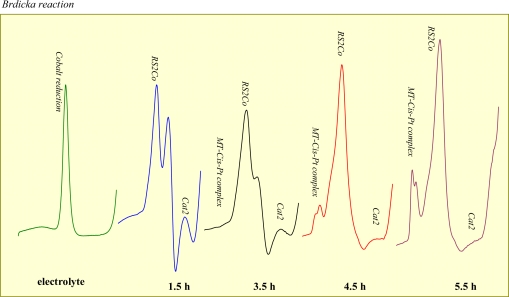
DP voltammograms of the rats′ blood treated with cisplatin for certain time intervals.

The presence of MT-cisplatin complex was confirmed also on the rats treated intraperitoneally with cisplatin. After cisplatin application, blood was sampled from the tail. Samples were prepared by heat treatment and analysed by the AdTS differential pulse voltammetry Brdickás reaction. Figure 4 shows DP voltammograms of blood obtained from rats in certain time intervals. It is clearly shown that the signal corresponding to the MT-*cis*Pt complex enhanced with increasing time after application. On the other hand Cat2 signal decreased. Cat2 is due to presence of free –SH moieties [49]. MT interacts with cisplatin via these moieties. Therefore, a decline of this signal correlates well with the fact that free thiol moieties of MT are saturated by cisplatin. This finding encouraged us to use other method to study the interaction of MT with cisplatin.

### 2.3. Detection of MT by quartz crystal microbalance

**Figure 5.**
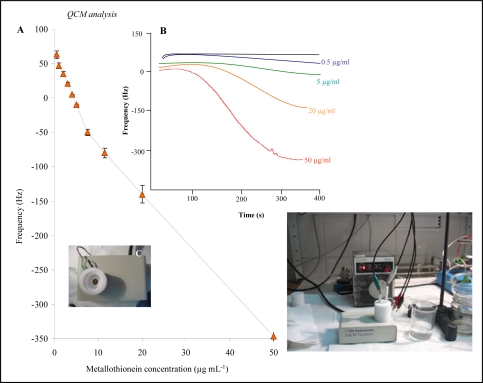
QCM analysis of MT on the gold crystal. (A) Dependence of the crystal frequency on MT concentration. (B) Typical QCM signals of frequency change after application of 0, 0.5, 5, 20 and 50 μg/mL MT. In inset: Photograph of experimental arrangement of QCM. Supporting electrolyte: 0.2 M phosphate buffer (pH 7.0), flow rate 1 mL min^−1^ for rinsing the crystal, n = 5.

**Figure 6.**
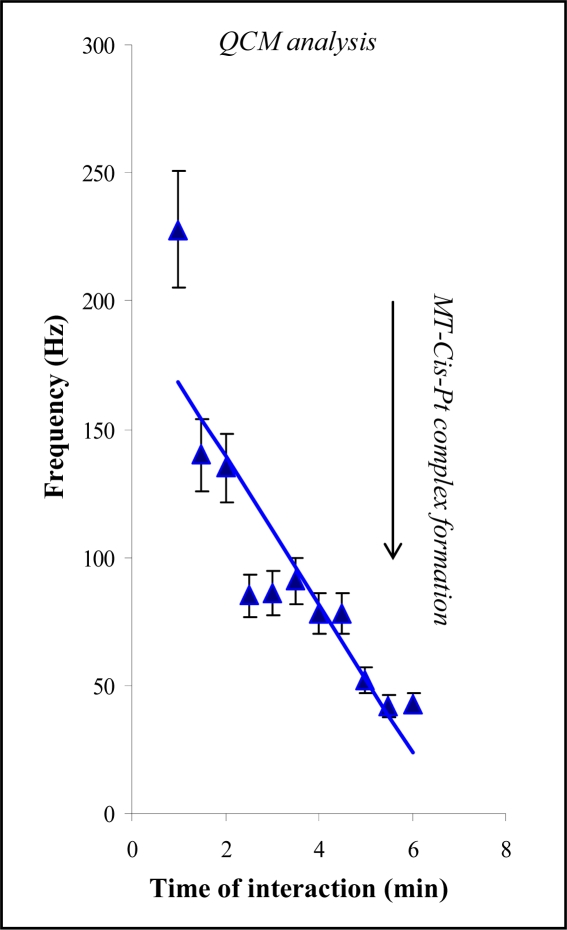
QCM analysis of MT-cisplatin interactions (various times of interaction (MT: 10 μg mL^−1^, cisplatin: 100 μg mL^−1^), n = 5.

In addition to mercury electrodes, MT is characterized by its high affinity for gold electrodes. Thus, we studied interaction of MT with a gold crystal by the changes in frequency of the crystal. The instrument used for this purpose is shown in Figure 5. A Teflon body containing a gold electrode was rinsed with supporting electrolyte with the use of a peristaltic pump. We observed changes in the frequency of the gold crystal with increasing MT concentration. The dependence obtained is shown in Figure 5A. Typical signals of the frequency change after introducing of 0, 0.5, 5, 20 and 50 μg mL^−1^ MT are shown in Figure 5B. We found that the frequency decreased as the MT concentration increased, according to the following equation: y = −8.0405x + 38.359 (R^2^ = 0.9746; R.S.D. = 4.5%). For analytical purposes we attempted to divide the concentration interval into two parts. The first part (concentration of MT = 0.5 – 7.5 μM) was strictly linear (y = −15.537x + 66.907; R^2^ = 0.9959). The second part (concentration of MT = 7.5 – 50 μM) had the following parameters: y = −6.9575x + 0.416; R^2^ = 0.9999; R.S.D. = 6.1%. We used the first part of this curve to determine the average change in frequency per 1 ng of MT. One ng of MT caused an approx. 3 Hz decrease in the frequency. Further, we used QCM to study the MT interaction with cisplatin. The experiment was carried out similarly as in case of HMDE. A gold electrode was modified by MT and immersed into a solution of cisplatin. The measured frequency decreased with as the interaction time increased (Figure 6). The highest decrease was determined after 90 s long MT-cisplatin interaction. Then, the signal decreased gradually.

**Figure 7.**
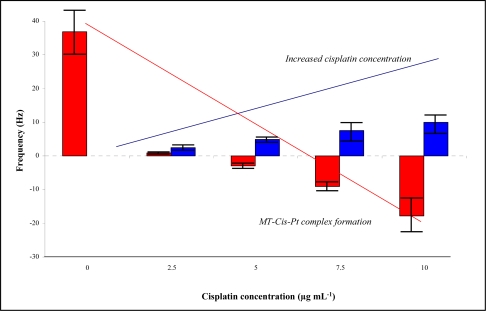
QCM analysis of MT-cisplatin interactions, n = 5.

Moreover the influence of cisplatin concentration on the frequency of the gold electrode modified by MT (10 μg mL^−1^) was studied. This dependence is shown in Figure 7. The blue columns represent amounts of the added cisplatin; the red columns show the changes in the frequency after cisplatin interaction. It follows from the results obtained that increasing cisplatin concentration resulted in the decrease in the frequency. Already after addition of 2.5 μg mL^−1^ cisplatin, the MT signal was reduced by more than 90 %. A concentration 10 μg mL^−1^ of cisplatin led to a 150% reduction of the signal.

Based on these results we attempted to calculate amount of cisplatin bonded to the MT-modified QCM. In the previous paragraph we noted that one ng of MT caused approx. a 3 Hz decrease in the frequency. The highest cisplatin concentration (50 ng) resulted in the frequency change of 54.65 Hz. This change shows that 18.33 ng of cisplatin is bonded to the MT-modified QCM. One molecule of MT is able to bind up to seven molecules of divalent heavy metal ion [60,61]. Platinum in cisplatin is divalent, so it may be suggested that MT should be able to bind seven molecules of cisplatin. It has been reported that cisplatin administration to rabbits yielded the species Pt_2_Zn_5_MT [62]. Nevertheless *in vitro* experiments showed that cisplatin has much more higher affinity to MT compared with cadmium(II) and zinc(II) ions [4,35]. Therefore we expected that cisplatin can saturate all binding sites of a MT standard containing cadmium(II) and zinc(II) only. The gold electrode was modified by 50 ng of MT with a theoretical capacity to bind with 17.5 ng of cisplatin. The difference between the experimentally estimated value and theoretical value can be related to unspecific interactions between the cysteine moieties of MT and cisplatin. Moreover we can conclude that cisplatin binds to the MT structure rapidly and tightly, because the signal corresponding to MT decreased even after interaction of a five times lower concentration of cisplatin, compared to that of MT. The association constant of platinum to MT KPt-MT = 2.3 × 10^−23^ was obtained, which is in very good agreement with the result derived from the hydrogen-platinum competition [4].

## 3. Materials and Methods

### 3.1. Chemicals, pH measurements and materials

Rabbit liver MT (MW 7143 g mol^−1^), containing 5.9 % Cd and 0.5 % Zn, was purchased from Sigma Aldrich (St. Louis, USA). The stock standard solutions of metallothionein (MT) was prepared with ACS water (Sigma-Aldrich, USA) and stored in the dark at −20 °C. Working standard solutions were prepared daily by dilution of the stock solutions with ACS water. The chemotherapeutic drug of cisplatin was synthesized and provided by Pliva-Lachema (Brno, Czech Republic). Stock standard solutions of cisplatin (500 μg mL^−1^) were prepared with sodium chloride solution (0.75 M, pH 5.0) and stored in the dark at −20 °C. The pH value was measured using WTW inoLab pH meter (Weilheim, Germany). Deionised water underwent demineralization by reverse osmosis using the instruments Aqua Osmotic 02 (Aqua Osmotic, Tisnov, Czech Republic) and then it was subsequently purified using Millipore RG (Millipore Corp., USA, 18 MΩ) – MiliQ water.

### 3.2. Preparation of biological samples for metallothionein determination

Blood samples were prepared by heat treatment. Briefly, the sample was kept at 99 °C in a thermomixer (Eppendorf 5430, USA) for 15 min. with occasional stirring, and then cooled to 4 °C. The denatured homogenates were centrifuged at 4 °C, 15,000g for 30 min. (Eppendorf 5402, USA). Heat treatment effectively denatures and removes high molecular weight proteins from samples [49].

### 3.3. Rats

Male Wistar rats (Faculty of Medicine, Masaryk University, Brno, Czech Republic), 8 weeks old (270–280 g), were used in our experiments. Six specimens were exposed to one dose of 1.05 mg of cisplatin per kg. Cisplatin was administered intraperitoneally.

### 3.4. Electrochemical measurements – Brdickás reaction

Electrochemical measurements were performed with an AUTOLAB Analyzer (EcoChemie, Netherlands) connected to a VA-Stand 663 (Metrohm, Switzerland), by using a standard cell with three electrodes. A hanging mercury drop electrode (HMDE) with a drop area of 0.4 mm^2^ was employed as the working electrode. The Ag/AgCl/3M KCl electrode was serving as the reference electrode. The glassy carbon electrode was used as the auxiliary electrode. The software GPES 4.9 supplied by EcoChemie was employed for the smoothing and baseline correction. The principle of the adsorptive transfer stripping technique (AdTS) is based on the strong adsorbing of the target molecule on the electrode surface at an open electrode circuit. The electrode is washed in the rinsing buffer. The electrode is further transferred to the supporting electrolyte and measured. The Brdicka supporting electrolyte containing 1 mM Co(NH_3_)_6_Cl_3_ and 1 M ammonia buffer (NH_3_(aq) + NH_4_Cl, pH = 9.6) was used and changed after five measurements; surface-active agent was not added. The samples of the MT were reduced before each measurement by 1 mM *tris*(2-carboxyethyl)phosphine addition according to [63]. AdTS DPV Brdickás reaction parameters were as follows: the initial potential of −0.35 V, the final potential −1.8 V, the modulation time 0.057 s, the time interval 0.2 s, the step potential of 1.05 mV, the modulation amplitude of 250 mV, E_ads_ = 0 V. All experiments were carried out at 4 °C (Julabo F12, Germany).

### 3.5. Quartz crystal microbalance

QCM measurements were performed by using 8-MHz ATcuts with gold electrodes on both sides (diameter 5 mm) supplied by CH Instruments (Austin, TX, USA). The output tube from the cell was connected to a peristaltic pump (Pump P-1, Biosciences, Amershan, UK). The measurement arrangement consisted of the QCM sensor fixed inside the flow-through cell and only one side was exposed to the flowing solutions. Connections were realized by using of the PVC tubing (internal diameter 0.15 mm). The crystals settled in the flow-through cell were connected to an oscillator circuit (International Manufacturing, Oklahoma City, USA) and the output frequency was determined using a universal counter (UZ 2400; UTES/Grundig, Brno, Czech Republic). Data were displayed and recorded by using of the CHI software (CHI, version 7.12, Austin, TX, USA).

### 3.6 Descriptive statistics

Data were processed by using the MICROSOFT EXCEL® (USA). Results are expressed as mean ± the standard deviation (S.D.) unless stated otherwise.

## 4. Conclusions

We have demonstrated that Brdickás reaction can be considered as a suitable tool for the study of the interactions of platinum-based therapeutics with metallothionein. The concentration of MT and cisplatin used in this study was similar to those found in the body, where the concentrations of these compounds ranges from sub-units to units μM [11,21,64,65]. Concerning our findings, we have discovered that a new signal appeared after MT-cisplatin interaction, which correlated well both with time of interaction and concentration of the drug. The formation of a MT-cisplatin complex was also confirmed in the experiments performed *in vivo* on the male rats which were treated with cisplatin. Interaction of MT with cisplatin was also investigated by QCM. It follows that MT-cisplatin interaction is relatively rapid and surely plays an important role in the therapy of malignant diseases. These results can contribute to simulation of the processes proceeding *in vivo* and may help during the proposal of therapy.
